# Supplementary material for: A bed nucleus of stria terminalis microcircuit regulating inflammation-associated modulation of feeding
Source: Nat Commun. 2019 Jun 24;10:2769. doi: 10.1038/s41467-019-10715-x (PMC6591327; doi:10.1038/s41467-019-10715-x)
Supplement: Supplementary file 1 — Supplementary Information [file 41467_2019_10715_MOESM1_ESM.pdf]

A bed nucleus of stria terminalis microcircuit regulating inflammation-associated modulation of feeding

Wang et al.

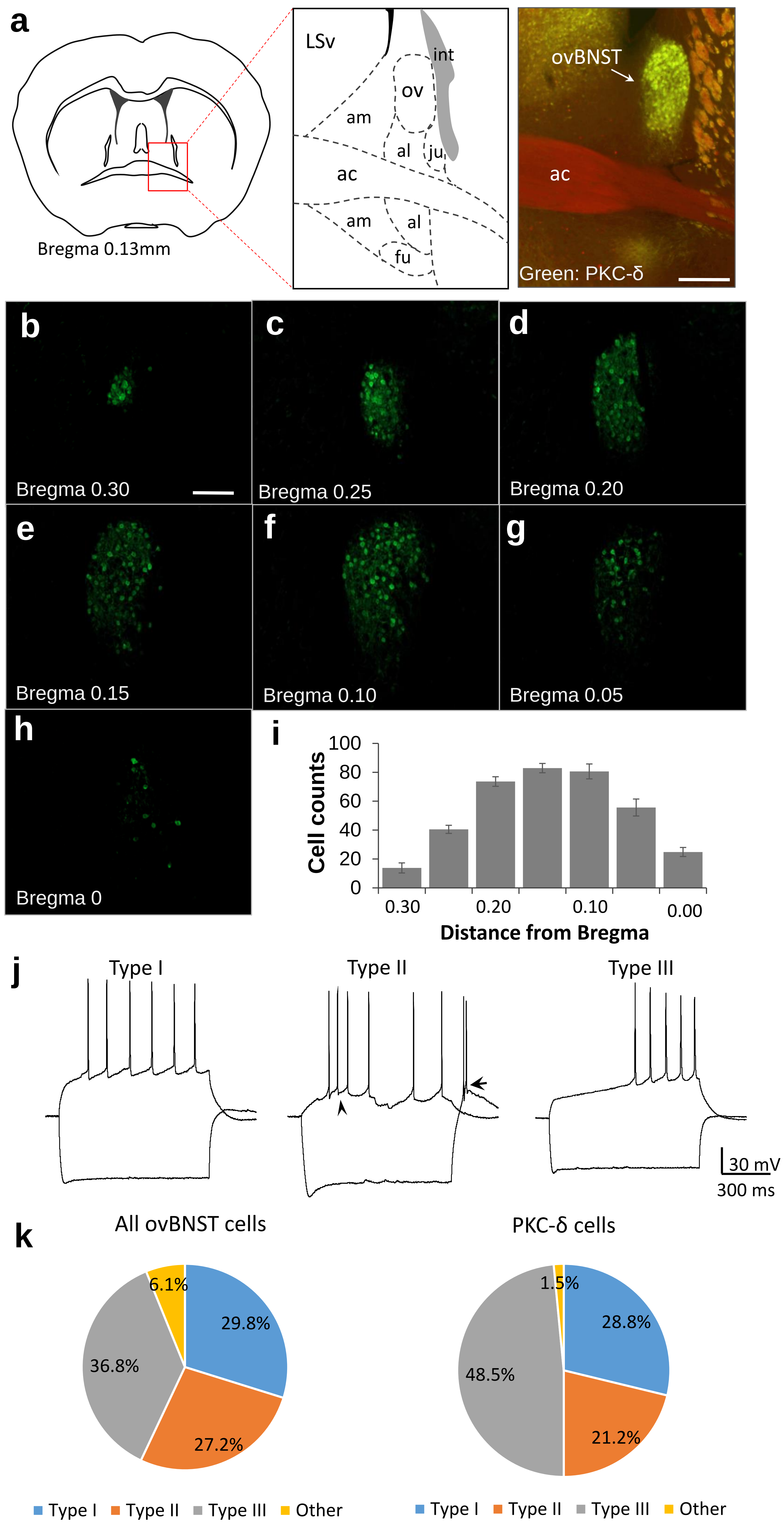

Supplementary Figure 1. **Characterization of ovBNST PKC-δ neurons**.
a. Immunostaining of the PKC-δ (green) shows that PKC-δ labels neurons in oval region of the BNST. The red background (red) is enhanced to show the BNST and surrounding areas. al, anterolateral BNST; am, anteromedial BNST; int, internal capsule; ju, juxtacapsular nucleus; LSv, ventral lateral septum. Scale bars, 200 µm.
b-h. Representative immunostaining images show that PKC-δ neurons are distributed from anterior to posterior ovBNST. Scale bar, 100 µm.
i. The number of PKC-δ neurons in ovBNST of different coronal sections throughout the anteroposterior axis. Data represent mean ± s.e.m. n = 5 animals.
j. Sample electrophysiological traces show three different types of ovBNST neurons in response to current injections. Type I cells (also called regular spiking cells) were characterized by a steady firing rate, but did not exhibit burst-firing activity. Type II cells (also called low-threshold bursting cells) exhibited rebound spiking (indicated by arrow) after the hyperpolarizing current steps or burst-firing activity (indicated by arrow head). Type III cells were classified based on a significant delay of firing in response to suprathreshold depolarizations. Cells do not fit into these three types are classified as Other type cells.
k. Relative frequency of the four types of cells in all ovBNST cells (n = 114 cells) and PKC-δ cells (n = 66 cells).
Source data are provided as a Source Data file Source data-Supplementary_Figs.

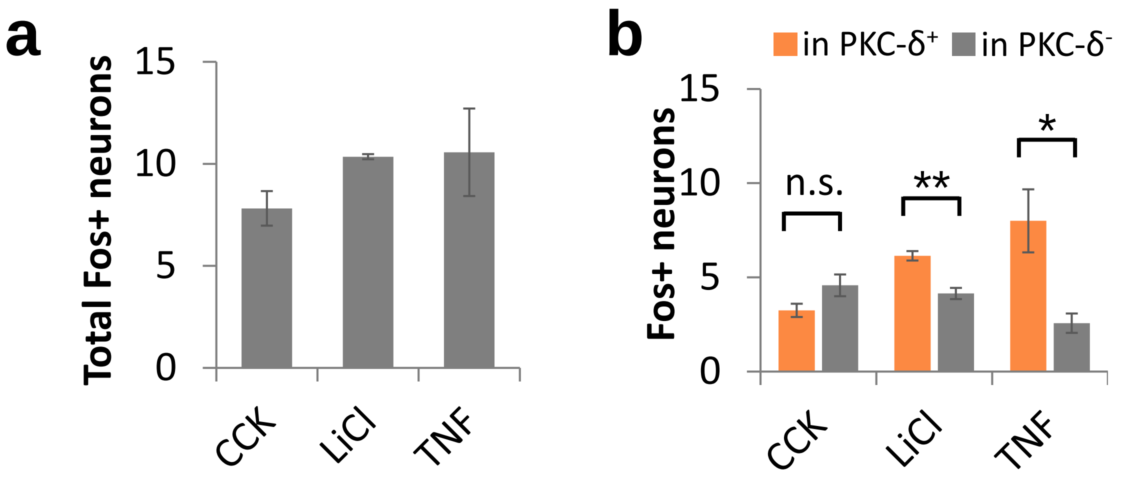

Supplementary Figure 2. Effect of CCK, LiCl or TNFα on ovBNST neurons.
a. Total number of c-Fos positive neurons in ovBNST after IP injection of CCK (5 μg/kg, Tocris), LiCl (150 mg/kg, Sigma, prepared in 150 mM), or TNFα (100 μg/kg, BD).
b. Fos positive neurons in PKC-δ+ or PKC-δ- population identified by immunostaining. Unpaired t-test (CCK, $t_{12} = 1.97$, $p = 0.072$; LiCl, $t_4 = 5.16$, $p = 0.0067$; TNFα, $t_6 = 3.1$, $p = 0.021$). n = 3-6 animals in each group. Data shown as mean ± s.e.m.
Source data are provided as a Source Data file Source data-Supplementary_Figs.

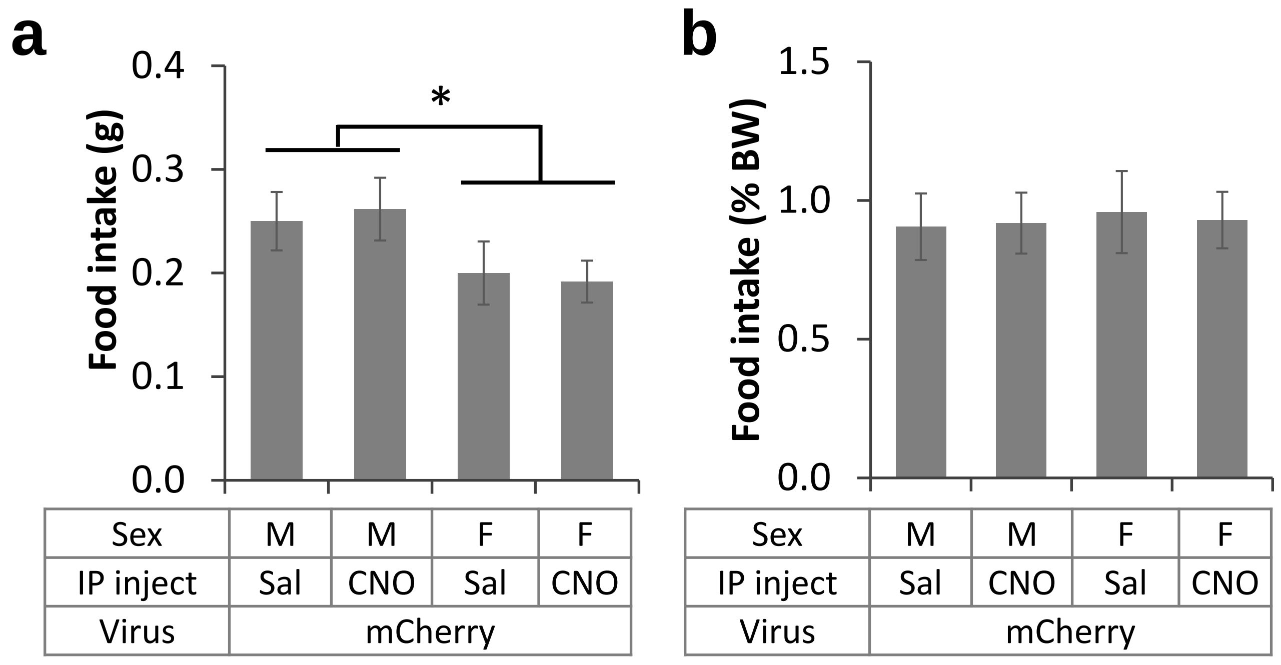

Supplementary Figure 3. IP injection of CNO does not affect food intake of control mice.
a. The amount of food intake is not affected by CNO or saline injection but shows a decreasing trend in female animals compared to male mice. Two-way ANOVA, $F_{(1, 20)} = 4.93$, $p = 0.038$. n = 6 animals in each group.
b. The food intake is not significantly different between male and female mice when normalized to their body weight (% BW). Two-way ANOVA, $F_{(1, 20)} = 0.07$, $p = 0.80$. n = 6 animals in each group. Data shown as mean ± s.e.m.
Source data are provided as a Source Data file Source data-Supplementary_Figs.

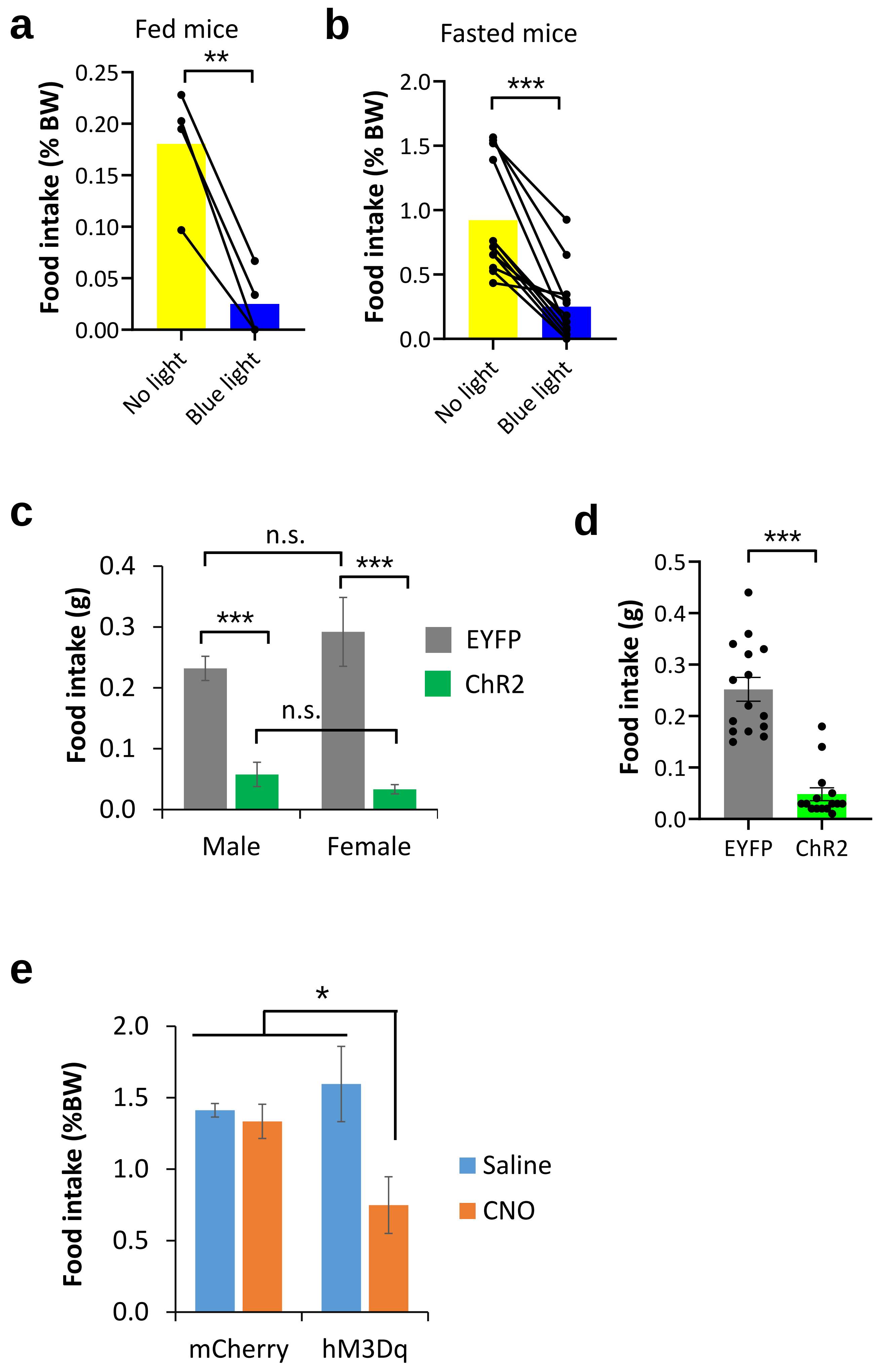

Supplementary Figure 4. Activation of ovBNST PKC-δ neurons suppresses food intake.

a, b. Food intake was suppressed in mice in which ovBNST PKC-δ neurons were activated by blue light (473 nm) in fed (**a**) and fasted status (**b**). Light pulse 10 ms pulse width, 15 Hz. Fed mice, food intake was measured in a 30-min feeding session, n = 4 animals in each group, paired *t*-test, $F_{(3)} = 7.11$, ** $p = 0.0057$. Fasted mice, food intake was measured in a 20-min feeding session, n = 12 animals in each group, paired *t*-test, $F_{(11)} = 6.50$, *** $p < 0.0001$.

c. Optogenetic activation of the ovBNST PKC-δ neurons suppresses food intake in both male and female mice. Mice are 24-hr fasted. Food intake was measured in a 20-min feeding session. Two-way ANOVA with post-hoc Bonferroni's *t*-test, $F_{(1, 25)} = 63.4$, *** $p < 0.001$, no significant difference between male and female groups, n = 10, 9, 5, 6 animals for Male-EYFP, Male-ChR2, Female-EYFP, Female-ChR2, respectively. Bar graph data shown as mean ± s.e.m.

d. The food intake from male and female animals were pooled together. Mice are 24-hr fasted. Food intake was measured in a 20-min feeding session. Unpaired *t*-test, $F_{(28)} = 7.78$, *** $p < 0.0001$, n = 15 animals in each group.

e. Chemogenetic activation of the ovBNST PKC-δ neurons suppresses food intake in 2-hr feeding session. Animals are fasted for 24 hours. 0.5 mg/kg CNO or equivalent volume of saline was injected 30-40 min before the feeding test. Two-way ANOVA with post-hoc Bonferroni's t test, $F_{(1, 16)} = 4.75$, $p < 0.05$. n =4, 4, 6, and 6 animals for mCherry-Saline, mCherry-CNO, hM3Dq-saline and hM3Dq-CNO, respectively. Data shown as mean ± s.e.m.

Source data are provided as a Source Data file Source data-Supplementary_Figs.

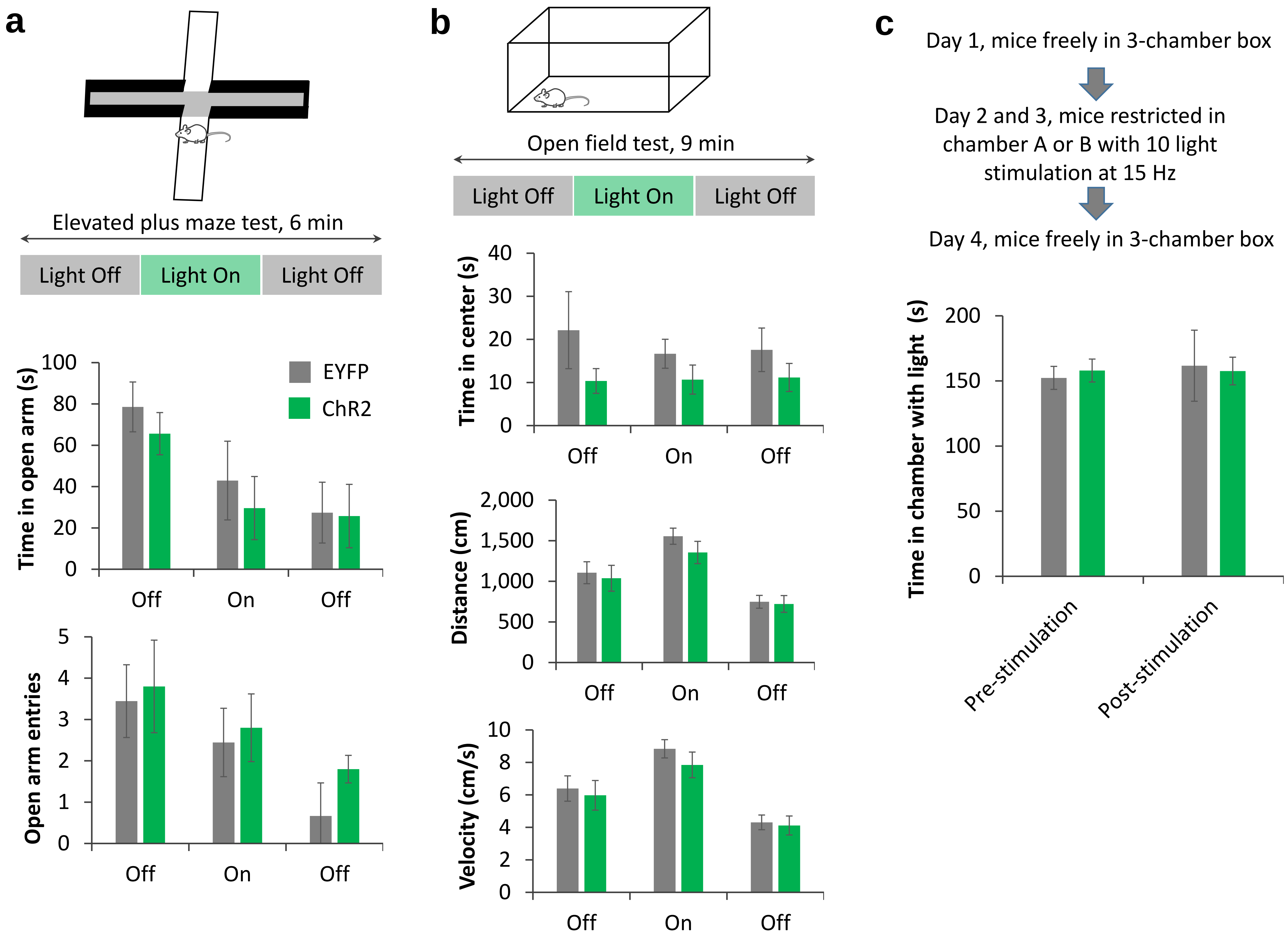

Supplementary Figure 5. Activation of the ovBNST-PKC-δ neurons on behaviors.

a-c. Optogenetic activation of the ovBNST PKC-δ neurons does not affect anxiety levels or mobility significantly in the elevated plus maze (**a**) or open field test (**b**), nor does it affect conditioned place aversion (**c**). Two-way ANOVA, $F_{(1, 51)} = 0.61$, $p = 0.44$ (**a**, time in open arm), $F_{(1, 51)} = 0.82$, $p = 0.37$ (**a**, open arm entry), $F_{(1, 51)} = 4.2$, $p = 0.05$ (**b**, time in center), $F_{(1, 51)} = 0.94$, $p = 0.34$ (**b**, distance travelled), $F_{(1, 51)} = 0.85$, $p = 0.36$ (**b**, velocity), n = 9 animals expressing EYFP and 10 animals expressing ChR2-EYFP. Two-way ANOVA, $F_{(1, 16)} = 0.01$, $p = 0.96$ (**c**), n = 5 animals in each group. Data shown as mean ± s.e.m.

Source data are provided as a Source Data file Source data-Supplementary_Figs.

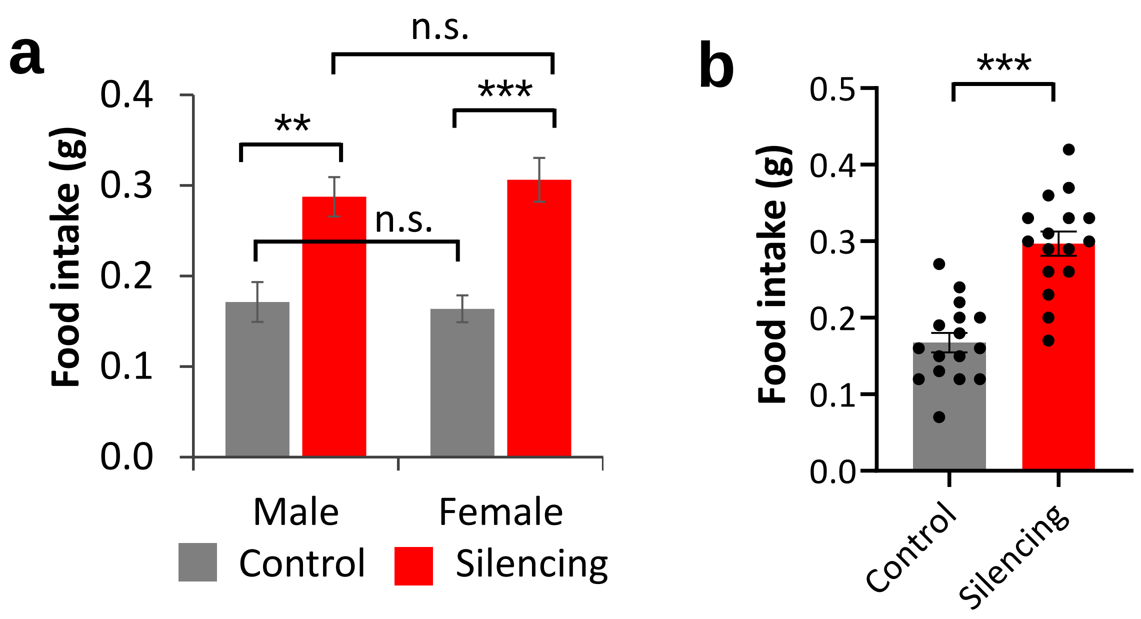

Supplementary Figure 6. Silencing ovBNST PKC-δ neurons increases food intake.

a. Chemogenetic silencing of the ovBNST PKC-δ neurons increases food intake in both male and female mice. Animals are 24-hr fasted, 5 mg/kg CNO or saline was injected 30-40 min before the feeding test. Feeding duration is 20 min. Two-way ANOVA with post-hoc Bonferroni's *t*-test, $F_{(1, 28)} = 37.9$, ** $p < 0.01$, *** $p < 0.001$, no significant difference between male and female groups, n = 8 animals in each group.

b. The food intake from male and female animals were pooled together. Mice are 24-hr fasted. Food intake was measured in a 20-min feeding session. Unpaired *t*-test, $F_{(30)} = 6.32$, *** $p < 0.0001$, n = 16 animals in each group. Data shown as mean ± s.e.m. Source data are provided as a Source Data file Source data-Supplementary_Figs.

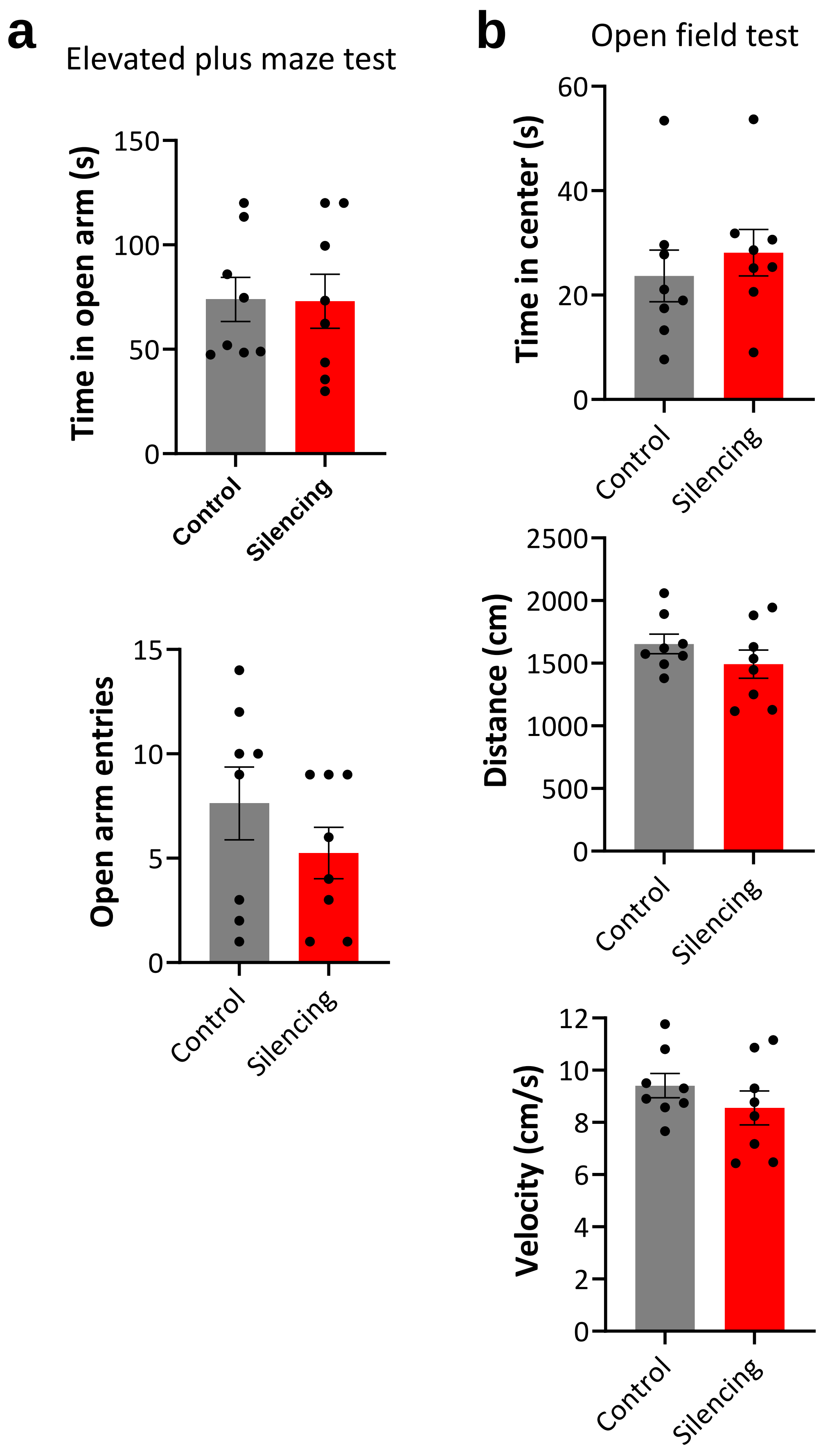

Supplementary Figure 7. Silencing of the ovBNST-PKC-δ neurons on anxiety.

a, b. Chemogenetic silencing of the ovBNST PKC-δ neurons does not affect anxiety levels significantly in elevated plus maze (**a**) and open field test (**b**). Unpaired *t*-test, t(14) = 0.052, p = 0.96 (**a**, time in open arm), t(14) = 1.11, p = 0.28 (**a**, open arm entry), t(14) = 0.67, p = 0.52 (**b**, time in center), t(14) = 1.18, p = 0.26 (**b**, distance travelled), t(14) = 1.07, p = 0.30 (**b**, velocity), n = 8 animals in each group. Data shown as mean ± s.e.m.

Source data are provided as a Source Data file Source data-Supplementary_Figs.

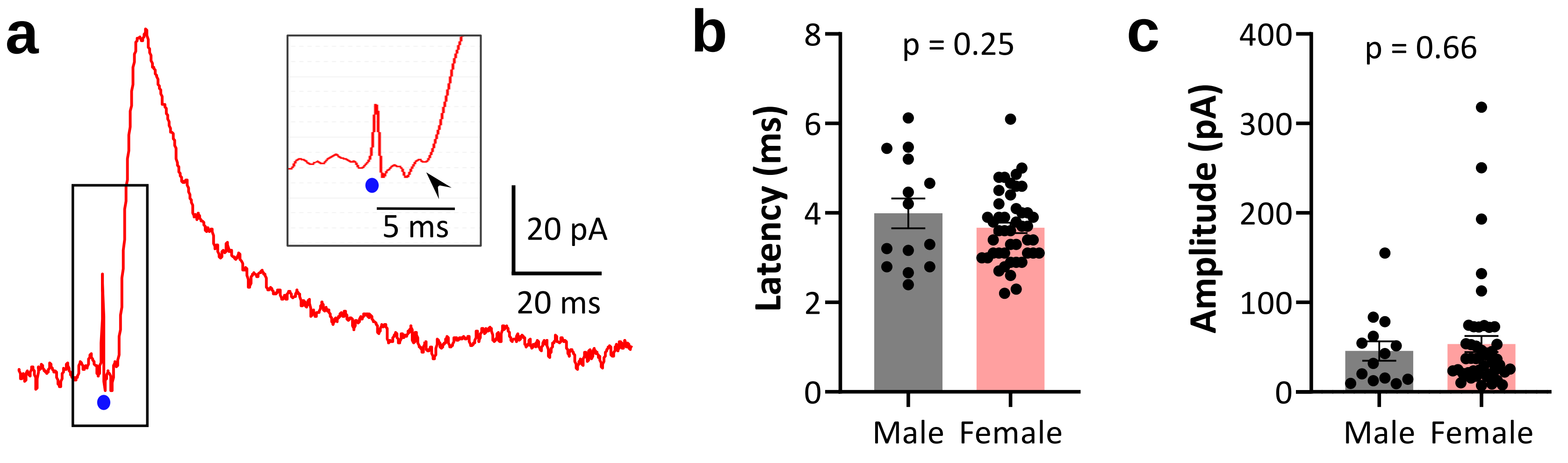

Supplementary Figure 8. **ovBNST PKC-δ neurons send monosynaptic inhibitory connections to vlBNST neurons**.

a. A sample electrophysiological recording trace shows that a monosynaptic IPSC in vlBNST neuron is triggered by light activation of the ovBNST PKC-δ neuron. Inset, latency of IPSC is measured from the light pulse to the start of IPSC (arrow head).

b, c. The IPSC latency (**b**) and amplitude (**c**) were not different between male and female mice. Unpaired *t*-test, t(58) = 1.16, p = 0.25 (latency), t(58) = 0.44, p = 0.66 (amplitude), n = 14 cells from 10 male mice, n = 46 cells from 28 female mice. Data shown as mean ± s.e.m.

Source data are provided as a Source Data file Source data-Supplementary_Figs.

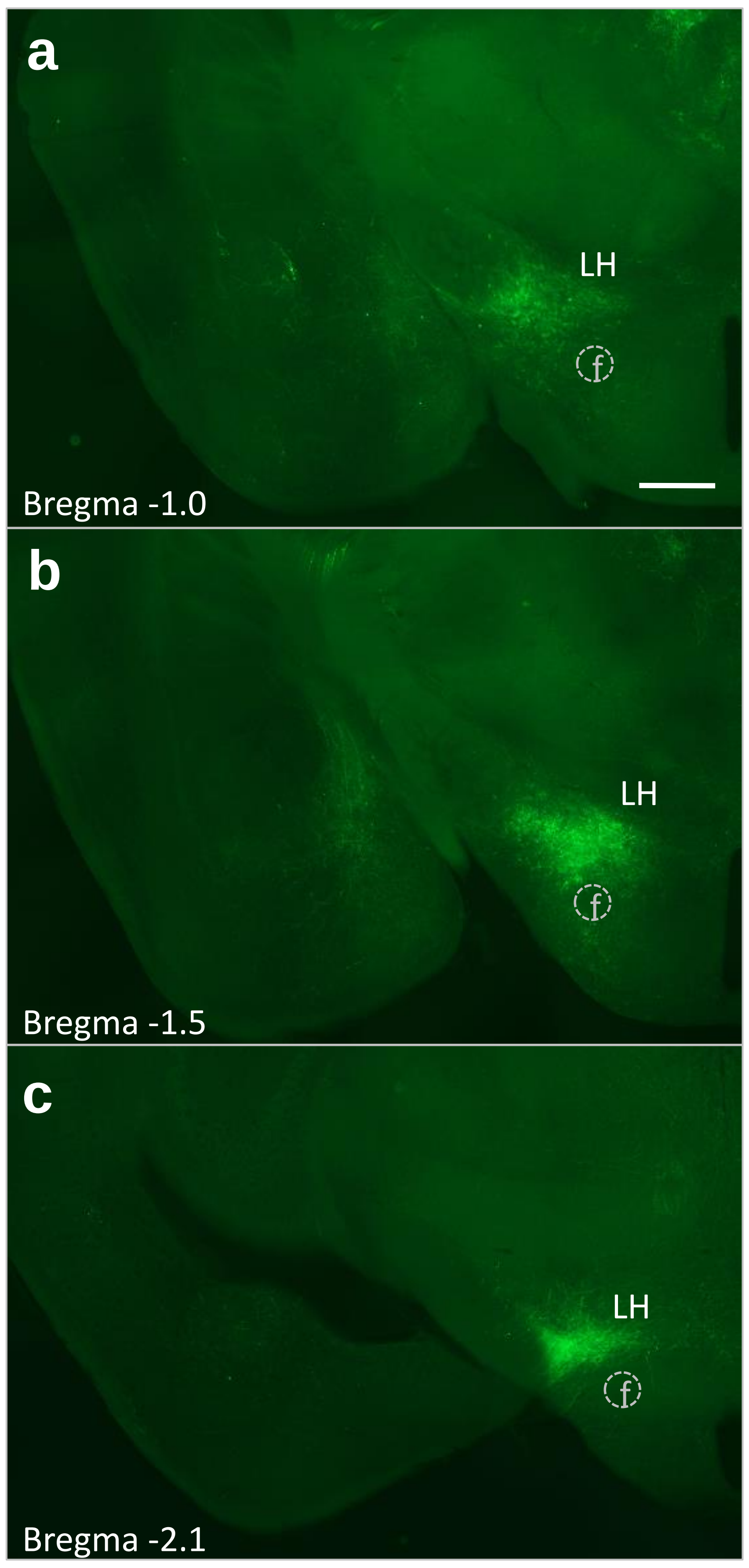

Supplementary Figure 9. **vlBNST neurons project to LH area**.
a-c. Representative images showing fluorescent nerve terminals from vlBNST neurons are distributed in a wide range of LH from anterior to posterior region. vlBNST neurons were labeled by co-injection of AAV-Flp and AAV-Flp^{ON}/Cre^{OFF}-ChR2-EYFP in vlBNST of the PKC-δ-Cre mice. Scale bar, 400 µm.

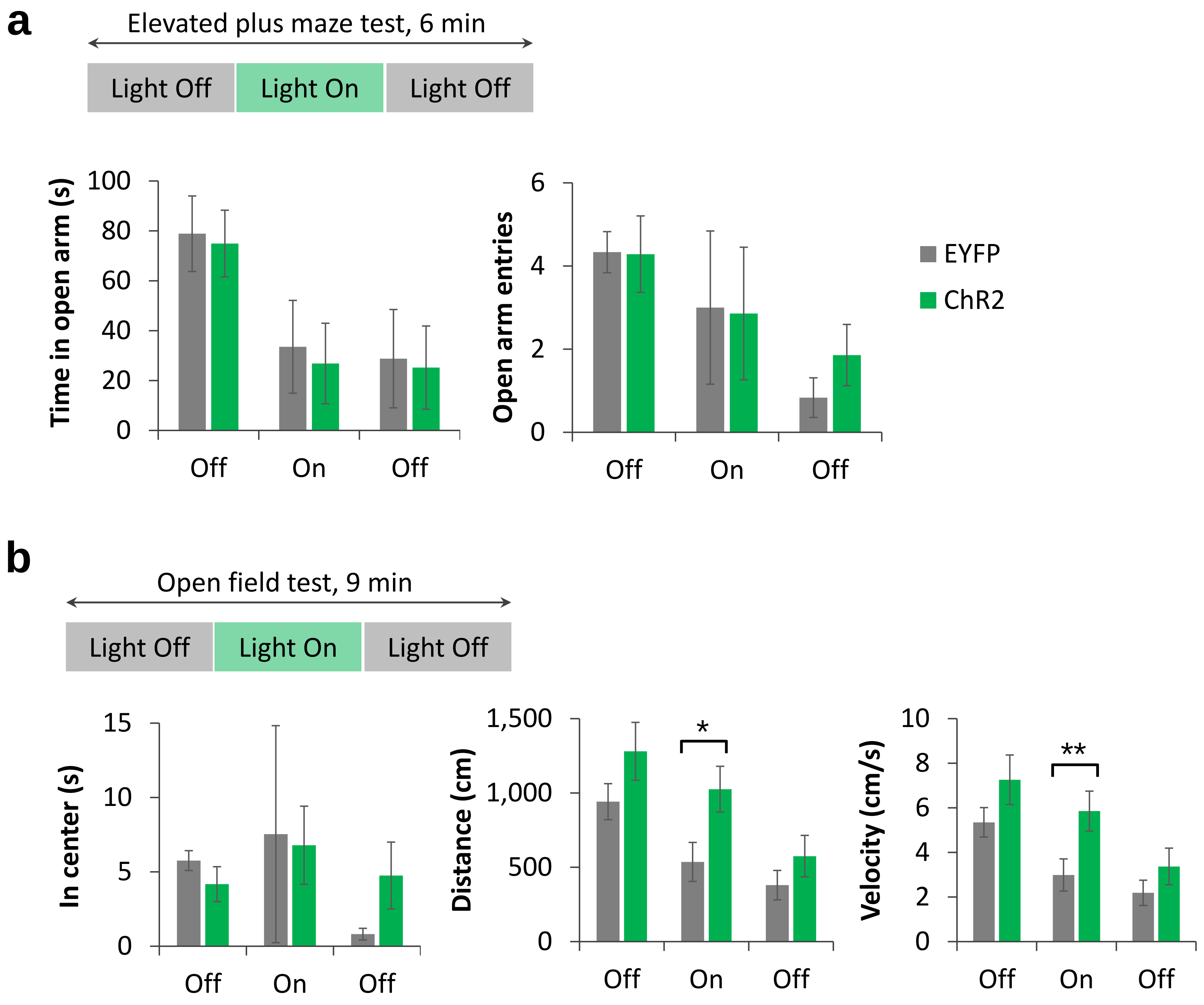

Supplementary Figure 10. **Activation of the LH-projecting vlBNST neurons in anxiety test.**
a, b. Optogenetic activation of the vlBNST-LH pathway does not affect anxiety levels or mobility significantly in the elevated plus maze (**a**) or open field test (**b**). However, there is a slight increase in the distance travelled and velocity upon light stimulation in open field test. Two-way ANOVA, $F_{(1, 33)} = 0.12$, $p = 0.44$ (**a**, time in open arm), $F_{(1, 33)} = 0.09$, $p = 0.77$ (**a**, open arm entry), $F_{(1, 33)} = 0.04$, $p = 0.84$ (**b**, time in center), $F_{(1, 33)} = 7.99$, $p = 0.01$ (**b**, distance travelled), $F_{(1, 33)} = 8.26$, $p = 0.007$ (**b**, velocity), n = 6 animals expressing EYFP and 6 animals expressing ChR2-EYFP. Data shown as mean ± s.e.m.
Source data are provided as a Source Data file Source data-Supplementary_Figs.

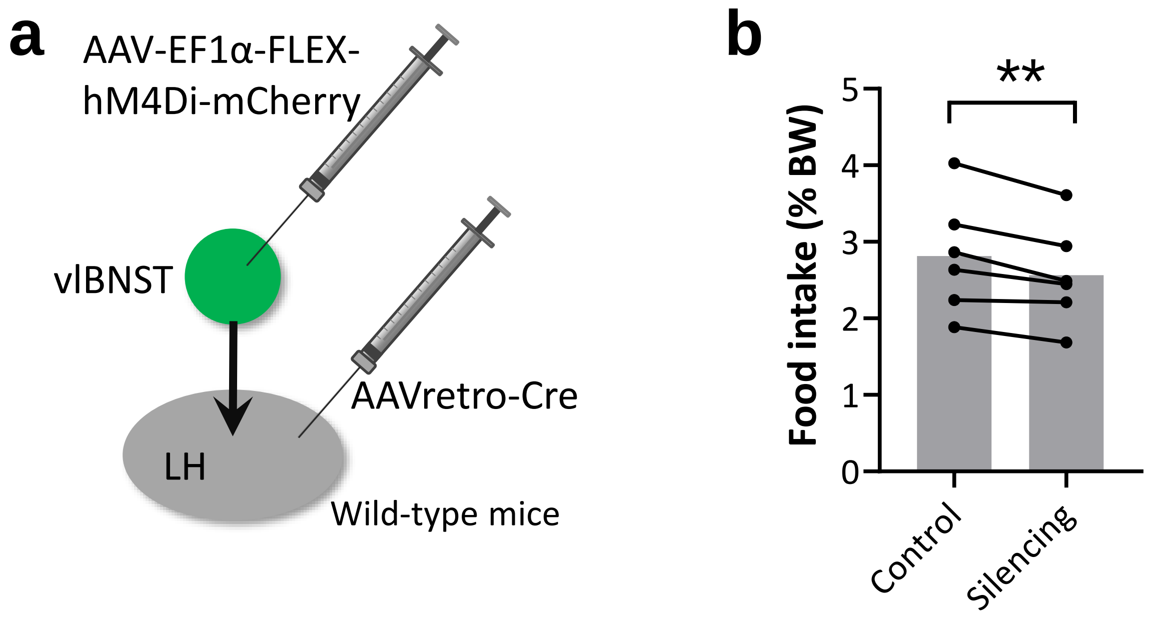

Supplementary Figure 11. **Silencing the LH-projecting vlBNST neurons suppresses food intake.**
a. Diagram shows the virus injection strategy to express hM4Di in LH-projecting vlBNST neurons.
b. Chemogenetic silencing the LH-projecting vlBNST neurons suppresses food intake. The food intake was measured in a feeding session of two hours. 5 mg/kg CNO or saline control was injected 30-40 min before the feeding test. n = 6 animals in each group, paired t-test, $t(5) = 4.346$. ** $p < 0.01$. Data bar graphs show means.
Source data are provided as a Source Data file Source data-Supplementary_Figs.

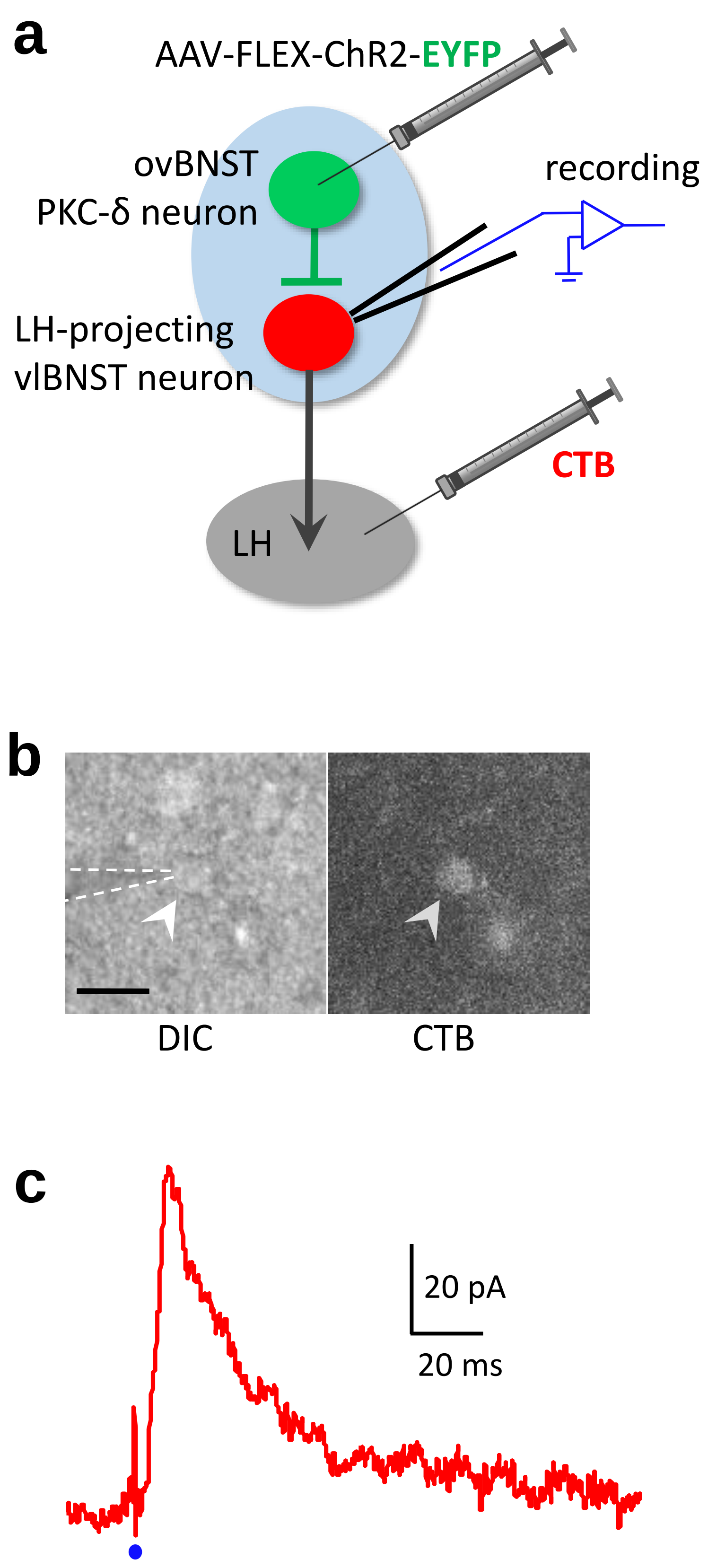

Supplementary Figure 12. **ovBNST PKC-δ neurons send monosynaptic inhibitory connections to LH-projecting vlBNST neurons**.
a. Diagram shows that ChR2 was expressed in ovBNST PKC-δ neurons and CTB was injected in LH area to back-label the LH-projecting vlBNST neurons.
b. CTB labelled vlBNST cell (arrowhead) is visualized in live brain slices. Scale bar, 10 µm.
c. Monosynaptic IPSC (latency, 3.8 ± 0.4 ms; amplitude, 48 ± 10 pA. mean ± s.e.m.; n = 4 cells) can be triggered in CTB-labelled vlBNST cells by light activation of the ovBNST PKC-δ neurons. Blue dot indicates a 2-ms light pulse.

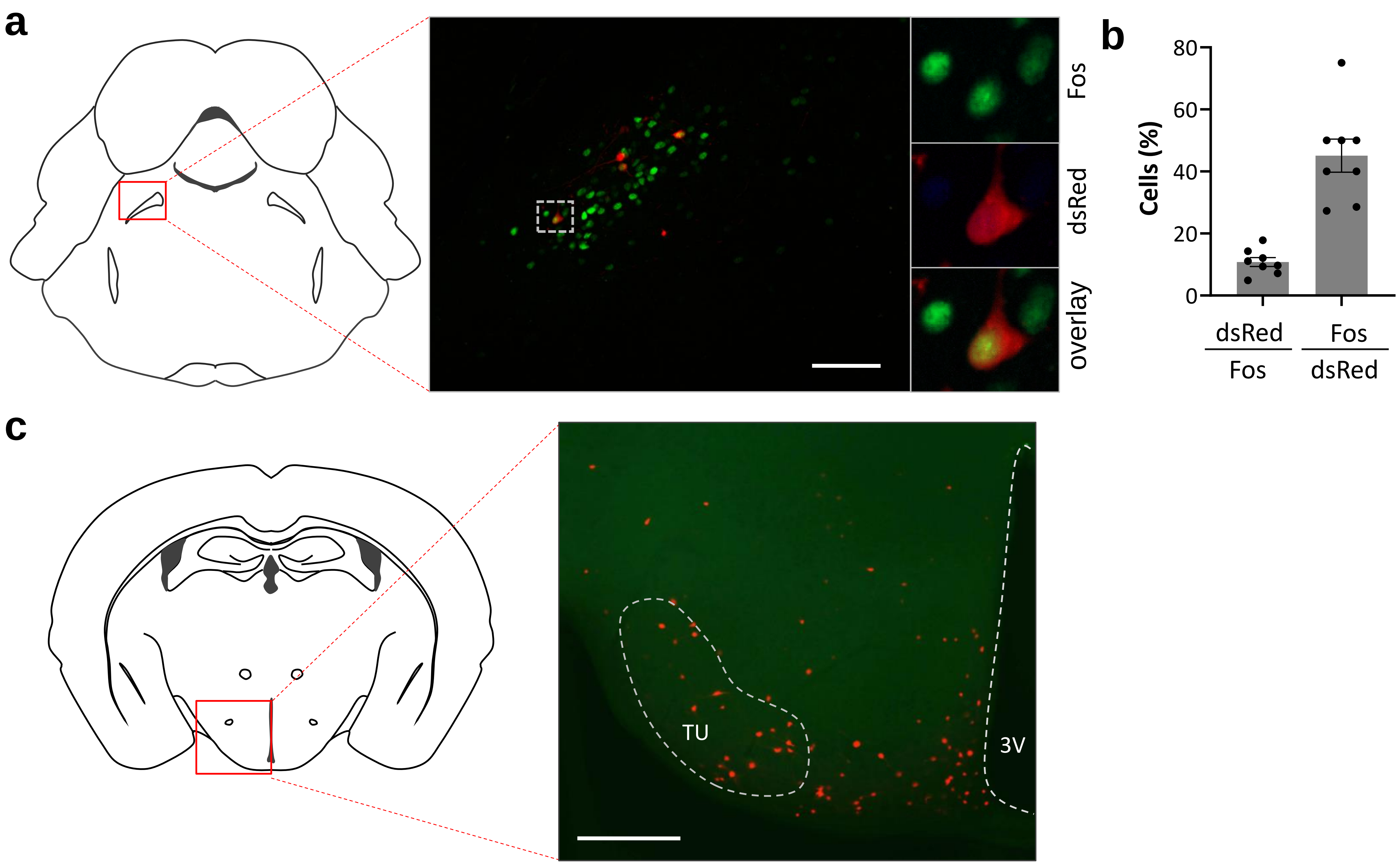

Supplementary Figure 13. Upstream inputs to ovBNST PKC-δ neurons.
a, b. LPB neurons that send monosynaptic innervation to ovBNST PKC-δ neurons are activated by IL-1β. Immunostaining (**a**) and quantification (**b**) of Fos immuno-like (green) and dsRed cells in LPB. Because the number of dsRed cells is less than 2% of the total number of LPB cells (estimated based on DAPI counting in the region with c-Fos expression) and the c-Fos expressing cells is less than 10%, more than 45% of the dsRed cells are positive for c-Fos staining is not a random overlap due to an increased c-Fos expression (the random overlap is <10%) but an actual overlap. Bar, 100 µm. Data were from n = 8 brain sections from 3 animals.
c. A representative image shows dsRed cells are found in tuberal nucleus region (TU). 3V, 3rd ventricle.
Scale bars, 200 µm. Data shown as mean ± s.e.m.
Source data are provided as a Source Data file Source data-Supplementary_Figs.
