# Supplementary material for: A bed nucleus of stria terminalis microcircuit regulating inflammation-associated modulation of feeding
Source: Nat Commun. 2019 Jun 24;10:2769. doi: 10.1038/s41467-019-10715-x (PMC6591327; doi:10.1038/s41467-019-10715-x)
Supplement: Supplementary file 3 — Reporting Summary [file 41467_2019_10715_MOESM3_ESM.pdf]

nature research

Corresponding author(s): Haijiang Cai

Last updated by author(s): Apr 8, 2019

Reporting Summary

Nature Research wishes to improve the reproducibility of the work that we publish. This form provides structure for consistency and transparency in reporting. For further information on Nature Research policies, see Authors & Referees and the Editorial Policy Checklist.

Statistics

For all statistical analyses, confirm that the following items are present in the figure legend, table legend, main text, or Methods section.

n/a	Confirmed	
☐	☒	The exact sample size (*n*) for each experimental group/condition, given as a discrete number and unit of measurement
☐	☒	A statement on whether measurements were taken from distinct samples or whether the same sample was measured repeatedly
☐	☒	The statistical test(s) used AND whether they are one- or two-sided<br>*Only common tests should be described solely by name; describe more complex techniques in the Methods section.*
☐	☒	A description of all covariates tested
☐	☒	A description of any assumptions or corrections, such as tests of normality and adjustment for multiple comparisons
☐	☒	A full description of the statistical parameters including central tendency (e.g. means) or other basic estimates (e.g. regression coefficient) AND variation (e.g. standard deviation) or associated estimates of uncertainty (e.g. confidence intervals)
☐	☒	For null hypothesis testing, the test statistic (e.g. *F*, *t*, *r*) with confidence intervals, effect sizes, degrees of freedom and *P* value noted<br>*Give P values as exact values whenever suitable.*
☒	☐	For Bayesian analysis, information on the choice of priors and Markov chain Monte Carlo settings
☒	☐	For hierarchical and complex designs, identification of the appropriate level for tests and full reporting of outcomes
☒	☐	Estimates of effect sizes (e.g. Cohen's *d*, Pearson's *r*), indicating how they were calculated

Our web collection on statistics for biologists contains articles on many of the points above.

Software and code

Policy information about availability of computer code

Data collection	A MATLAB based in-house behavioral annotation script is available from the corresponding author on reasonable request.
Data analysis	Data were analyzed with GraphPad Prism 8 Software.

For manuscripts utilizing custom algorithms or software that are central to the research but not yet described in published literature, software must be made available to editors/reviewers. We strongly encourage code deposition in a community repository (e.g. GitHub). See the Nature Research guidelines for submitting code & software for further information.

Data

Policy information about availability of data

All manuscripts must include a data availability statement. This statement should provide the following information, where applicable:

- Accession codes, unique identifiers, or web links for publicly available datasets
- A list of figures that have associated raw data
- A description of any restrictions on data availability

The complete datasets of the study are available from the corresponding author on reasonable request.

Field-specific reporting

Please select the one below that is the best fit for your research. If you are not sure, read the appropriate sections before making your selection.

☒ Life sciences ☐ Behavioural & social sciences ☐ Ecological, evolutionary & environmental sciences

For a reference copy of the document with all sections, see nature.com/documents/nr-reporting-summary-flat.pdf

Life sciences study design

All studies must disclose on these points even when the disclosure is negative.

Sample size	Sufficient sample sizes were chosen based on previous research in mice, as previously published in Nature Neuroscience from the author (Cai et al., 2014).
Data exclusions	Data exclusion criteria included lack of accurate stereotaxic targeting and/or viral expression and/or antibodies staining.
Replication	To ensure experimental findings can be easily reproduced, we included detailed methods and sources of all reagents and protocols for experiments included in the manuscript.
Randomization	Mice were randomly assigned to experimental groups, and all groups consisted of age- and sex-matched littermates.
Blinding	Experimenters were blinded to the viral treatment groups during data acquisition.

Reporting for specific materials, systems and methods

We require information from authors about some types of materials, experimental systems and methods used in many studies. Here, indicate whether each material, system or method listed is relevant to your study. If you are not sure if a list item applies to your research, read the appropriate section before selecting a response.

Materials & experimental systems

n/a	Involved in the study	
☐	☒	Antibodies
☒	☐	Eukaryotic cell lines
☒	☐	Palaeontology
☐	☒	Animals and other organisms
☒	☐	Human research participants
☒	☐	Clinical data

Methods

n/a	Involved in the study	
☒	☐	ChIP-seq
☒	☐	Flow cytometry
☒	☐	MRI-based neuroimaging

Antibodies

Antibodies used	Goat anti-c-Fos, polyclonal, Santa Cruz Biotech, sc-52-G;<br>Rabbit anti-PKC delta, monoclonal Abcam, ab182126;<br>Rabbit anti-AGRP, Phoenix Pharmaceuticals, 01765-3;<br>Goat anti-CGRP, polyclonal, Abcam, Ab36001;<br>Alexa Fluor 488 Donkey Anti-Goat IgG (H+L), Jackson ImmunoResearch, 705-545-003;<br>Alexa Fluor 488 Donkey Anti-Rabbit IgG (H+L), Jackson ImmunoResearch, 711-545-152;<br>Alexa Fluor 594 Donkey Anti-Goat IgG (H+L), Jackson ImmunoResearch, 705-585-003;<br>Alexa Fluor 594 Donkey Anti-Rabbit IgG (H+L), Jackson ImmunoResearch, 711-585-152;<br>Alexa Fluor 405 Goat Anti-Rabbit IgG (H+L), Thermo-Fisher; A-31556.
Validation	All antibodies used in the study are validated for species by manufacturer.

Animals and other organisms

Policy information about studies involving animals; ARRIVE guidelines recommended for reporting animal research

Laboratory animals	To ensure that the mice we used in this project have a consistent genetic background, we crossed the PKC-δ-Cre mice with the wild-type C57BL/6 mice from the Charles River Laboratory for at least 5 to 6 generations. The genotype of transgenic PKC-δ-Cre mice offspring is identified by PCR on genomic tail DNA. Both wild-type and PKC-δ-Cre offspring were used in this study. Survival surgery were performed when mice are 2-3 months old and behavioral tests were performed when mice are 3-5 months old. Both male and female mice were used in the study.
Wild animals	N/A
Field-collected samples	N/A
Ethics oversight	All animal care and experimental procedures were strictly conducted according to the guidelines of US National Institutes of Health for animal research and were approved by the Institutional Animal Care and Use Committee (IACUC) at the University of Arizona.

Note that full information on the approval of the study protocol must also be provided in the manuscript.
